# Metformin ameliorates olanzapine-induced obesity and glucose intolerance by regulating hypothalamic inflammation and microglial activation in female mice

**DOI:** 10.3389/fphar.2022.906717

**Published:** 2022-10-12

**Authors:** Sang Bum Suh, Nayoung Lee, Jaedeok Kim, Saeha Kim, Sooyeon Jang, Jong Kook Park, Keunwook Lee, Soo Young Choi, Hyung-Joo Kwon, Chan Hee Lee

**Affiliations:** ^1^ University of Ulsan College of Medicine, Seoul, South Korea; ^2^ Department of Biomedical Science, Hallym University, Chuncheon, South Korea; ^3^ Department of Microbiology, College of Medicine, Hallym University, Chuncheon, South Korea; ^4^ Program of Material Science for Medicine and Pharmaceutics, Hallym University, Chuncheon, South Korea

**Keywords:** second-generation antipsychotic drug, olanzapine, metformin, hypothalamus, microglia, leptin resistance, metabolic disease

## Abstract

Olanzapine (OLZ), a widely used second-generation antipsychotic drug, is known to cause metabolic side effects, including diabetes and obesity. Interestingly, OLZ-induced metabolic side effects have been demonstrated to be more profound in females in human studies and animal models. Metformin (MET) is often used as a medication for the metabolic side effects of OLZ. However, the mechanisms underlying OLZ-induced metabolic disturbances and their treatment remain unclear. Recent evidence has suggested that hypothalamic inflammation is a key component of the pathophysiology of metabolic disorders. On this background, we conducted this study with the following three objectives: 1) to investigate whether OLZ can independently induce hypothalamic microgliosis; 2) to examine whether there are sex-dependent differences in OLZ-induced hypothalamic microgliosis; and 3) to examine whether MET affects hypothalamic microgliosis. We found that administration of OLZ for 5 days induced systemic glucose intolerance and hypothalamic microgliosis and inflammation. Of note, both hypothalamic microglial activation and systemic glucose intolerance were far more evident in female mice than in male mice. The administration of MET attenuated hypothalamic microglial activation and prevented OLZ-induced systemic glucose intolerance and hypothalamic leptin resistance. Minocycline, a tetracycline derivative that prevents microgliosis, showed similar results when centrally injected. Our findings reveal that OLZ induces metabolic disorders by causing hypothalamic inflammation and that this inflammation is alleviated by MET administration.

## 1 Introduction

Olanzapine (OLZ) is widely used for the treatment of schizophrenia. It effectively controls schizophrenia by lowering hyperactive mesolimbic dopamine activity and increasing the dopamine sensitivity of the prefrontal cortex (Peuskens et al., 2005; Brisch et al., 2014). However, second-generation antipsychotics (APs), including OLZ, have serious metabolic side effects, and these side-effects are much more pronounced in women (Albaugh et al., 2006; Nasrallah, 2008; Bak et al., 2014). These drugs induce metabolic dysregulations, such as diabetes and obesity, which shorten the life span of schizophrenia patients by up to 10–20 years compared to that of those without schizophrenia (Tiihonen et al., 2009). Although the medications used for diabetes and obesity, such as metformin (MET), have been prescribed with APs to stop the progression and development of metabolic disorders (Baptista et al., 2007; de Silva et al., 2015; de Silva et al., 2016), the mechanisms underlying these effects remain largely unknown.

Evidence accumulated over the last few decades shows that hypothalamic inflammation is a critical component of the pathophysiology of metabolic dysregulation and obesity (Valdearcos et al., 2015; Jais and Brüning, 2017; Lee et al., 2018). Moreover, previous studies have shown that preventing hypothalamic microglial activation alleviates high-fat diet (HFD)-induced hypothalamic inflammation and leptin resistance, which implies that hypothalamic microglia are important therapeutic targets for obesity-related metabolic disorders (Valdearcos et al., 2014; André et al., 2017; Valdearcos et al., 2017; Lee et al., 2018). Under inflammatory conditions, activated microglia produce pro-inflammatory cytokines and enhance the expression of leptin resistance-related genes, such as protein tyrosine phosphatase 1B (*Ptp1b*) and suppressor of cytokine signaling 3 (*Socs3*) in hypothalamic neurons (Picardi et al., 2008; Pedroso et al., 2014). Despite the importance of hypothalamic microgliosis in the development of obesity and metabolic diseases, the central pathophysiology of OLZ-induced metabolic dysregulation still remains unclear.

In this study, we aimed to determine whether hypothalamic inflammation is induced independently by OLZ and whether the protective effect of MET is mediated by the regulation of hypothalamic inflammation and microgliosis. We investigated the changes in hypothalamic microglia in OLZ- or OLZ + MET-administered female and male mice. Hypothalamic inflammatory markers as well as hypothalamic response to leptin were measured after short-term OLZ administration or OLZ and MET coadministration. Our findings suggest that OLZ induces hypothalamic inflammation and metabolic disorders, which are ameliorated by MET through the regulation of hypothalamic microgliosis.

## 2 Materials and methods

### 2.1 Animals

All animal procedures were approved by the Institutional Animal Care and Use Committee (IACUC) of Hallym University (Hallym 2021-58). Seven-week-old C57BL/6J male and female mice were purchased from DBL (Chungbuk, Korea). Animals were singly-housed in a temperature-controlled room (22 ± 1°C) with a 12 h light-dark cycle (lights on 8 a.m.). Mice were freely provided either 45% HFD or chow diet (CD) as well as water ad libitum. Body weight and food intake were measured weekly and the daily food intake (kcal) was estimated by dividing weekly consumption by 7.

### 2.2 Drugs and experimental design

**FIGURE 1:**
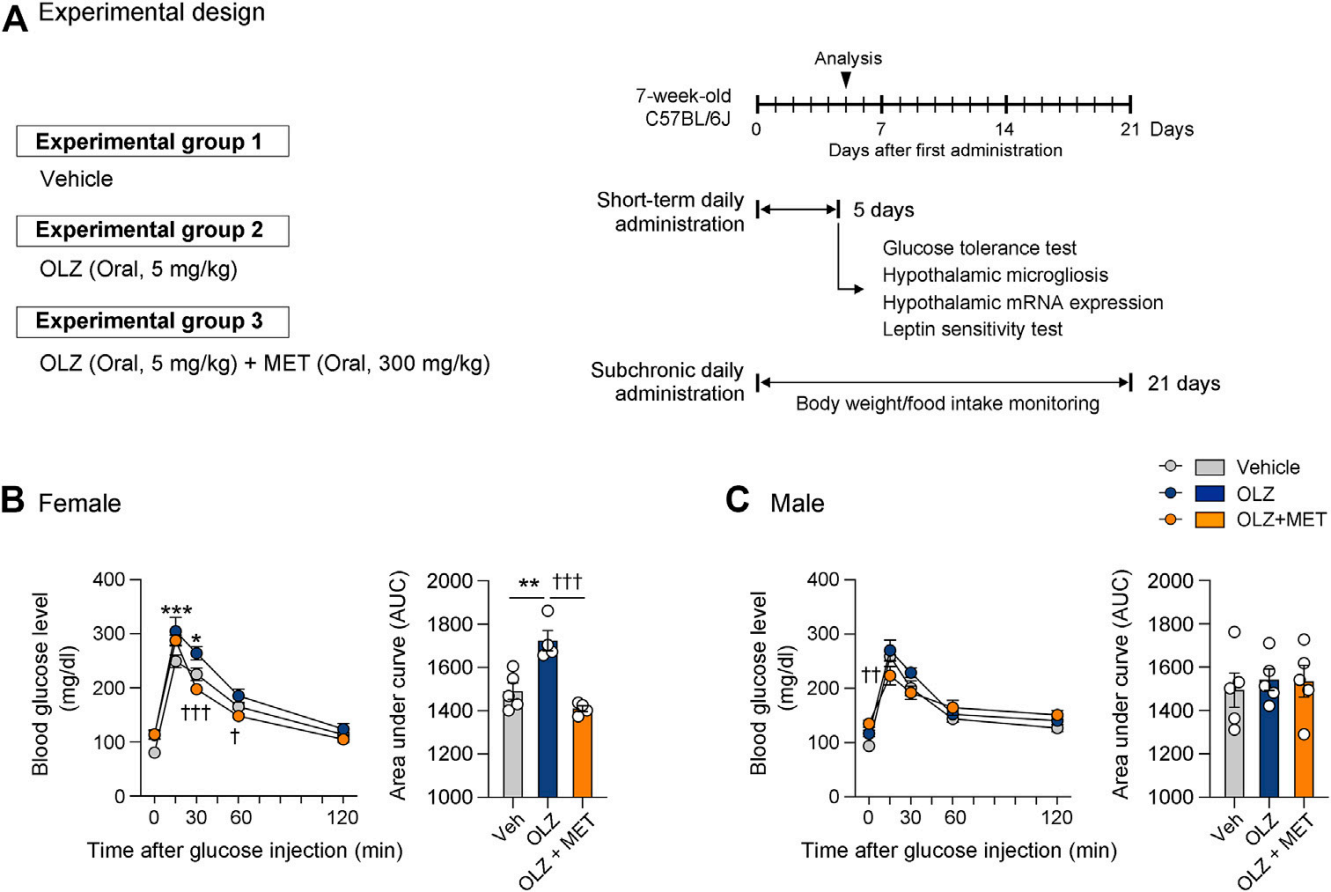
Metformin (MET) attenuates olanzapine (OLZ)-induced glucose intolerance in female mice. **(A)** Experimental scheme pertaining to mice that were administered OLZ or OLZ + MET on postnatal week 7. **(B,C)** Glucose tolerance tests were performed after the administration of OLZ or OLZ + MET for 5 days in female **(B)** and male mice **(C)** (*n* = 4–5). Arrows indicate measurement time points. Data are presented as mean ± SEM values. Data were analyzed by one-sided one-way analysis of variance (ANOVA) followed by a post hoc least significant difference (LSD) test. *p < 0.05 and ***p < 0.001 between Vehicle and OLZ groups, ^†^ *p* < 0.05, ^††^ *p* < 0.01 and ^†††^ *p* < 0.001 between OLZ and OLZ + MET groups.

OLZ (Sigma, #O1141), MET (Sigma, #PHR1084), and minocycline (MINO) (Sigma, #M9511) were purchased from Sigma-Aldrich. OLZ was used for antipsychotic drug-induced weight gain (AIWG) and dysglycemia experiments. OLZ dosage (5 mg/kg/d) and administration period were selected based on previous studies (Fernø et al., 2011; Ikegami et al., 2013; Lord et al., 2017; Castellani et al., 2018). OLZ was dissolved in dimethyl sulfoxide and subsequently diluted in normal saline (0.9% sodium chloride) to obtain the desired final concentration. MET was dissolved in sterile normal saline. The dosage for MET (300 mg/kg/d) was selected based on previous studies (Shore et al., 2008; Matsui et al., 2010; Calixto et al., 2013). Control mice were orally administered 10% dimethyl sulfoxide or normal saline. Animals in the Vehicle, OLZ, and MET groups received daily treatments by oral gavage between 8:00 a.m. and 9:00 a.m. MINO has been used as a drug to prevent AP-induced side effects in previous studies (Perez-Gomez et al., 2018; Zapata et al., 2020). MINO was dissolved in distilled water and subsequently diluted in normal saline to obtain the desired final concentration. The concentration of MINO that suppressed hypothalamic microgliosis while having no effect on food intake (Supplementary Figure S4) was selected for further animal experiments. MINO (5 μg) was injected via the intracerebroventricular (ICV) route 1 h before OLZ administration. Body weight and food intake monitoring were conducted for 21 days, and a glucose tolerance test, hypothalamic microgliosis analysis, hypothalamic mRNA expression analysis, and leptin sensitivity test were conducted on the 5th day of drug administration. Schematic representations of our experimental design are presented in Figures 1A, 4A, 5B.

### 2.3 Oral glucose tolerance test

We performed an oral glucose tolerance test on mice administered either OLZ or OLZ + MET for 5 days. For the glucose tolerance test, D-glucose (1 g/kg, Sigma, #G8270) was administered orally in overnight-fasted mice. Blood samples were obtained from the tail tip for measuring glucose level immediately before and 15, 30, 60, 90, and 120 min after injection. Glucose levels were measured using a glucometer (ACCU-CHEK^®^, Aviva Plus System).

### 2.4 Immunofluorescence Staining

Histological analysis was performed on the 5^th^ day of the vehicle, OLZ, or OLZ + MET administration. Isoflurane inhalations were used for mouse anesthesia. The mice were then transcardially perfused with 50 ml of cold saline and 50 ml of 4% paraformaldehyde. Whole brains were collected and fixed with 4% paraformaldehyde for 16 h at 4°C. Subsequently, the brains were placed in a dehydration solution (30% sucrose in phosphate-buffered saline (PBS)) for 48 h. The hypothalamic area of the brain was sectioned coronally every 30 μm using a cryostat (Leica, Wetzlar, Germany). The sections were stored at −70°C. For ionized calcium-binding adaptor protein-1 (Iba1) staining, hypothalamic slices were permeabilized in 0.5% PBS with Triton X-100 for 5 min and blocked with 3% donkey serum in 0.1% PBS with Triton X-100 at room temperature for 1 h. The slices were incubated with anti-mouse Iba1 antibody (1:400, Abcam, #ab5076) in blocking solution at 4°C for 16 h and then at room temperature (RT) for 1 h. For tumor necrosis factor (TNF)α staining, hypothalamic slices were blocked with 3% bovine serum albumin in 0.5% PBS with Triton X-100 at RT for 1 h and subsequently incubated with anti-mouse TNFα antibody (1:200, Abcam, #ab1793) in PBS at 4°C for 16 h and then at RT for 1 h. For c-Fos staining, hypothalamic slices were blocked with 3% bovine serum albumin (BSA) in 0.5% PBS with Triton X-100 at room temperature for 1 h and incubated with anti-c-Fos antibody (1:1000, Synaptic System, #226 008) in blocking solution at 4°C for 48 h. After washing with PBS, the hypothalamic slices were incubated with the appropriate Alexa-Flour 488-, 546-, or 555-conjugated secondary antibodies (1:1000, Invitrogen) at room temperature for 1 h. Confocal microscopy (Carl Zeiss #710, Germany) was employed for the observation of the fluorescent antibodies.

### 2.5 Hypothalamic gene expression

Hypothalamic gene expression was analyzed in mice administered OLZ or OLZ + MET for 5 days. The method for isolating the mediobasal hypothalamus was based on the previous studies (Kim et al., 2006; Han et al., 2014; Lee et al., 2018; Kang et al., 2021). Briefly, mice with ad libitum access to food were sacrificed by decapitation. The brains were flipped over and cut coronally before and after the protrusion of the median eminence into 1 mm-thick sections; a mouse brain matrix was used to facilitate this process (RWD, #68707). After laying the brain slide on its side, the ventral equilateral triangle region with a vertex at ½ point of the third ventricle (3V) was obtained. The tissues were quickly frozen using liquid nitrogen and preserved in a −70°C deep freezer. Total RNA was extracted from brain tissue using TRIzol (Life Technologies, #15596018) according to the manufacturer’s protocol. The RNA was reverse-transcribed to generate cDNA. The mRNA expression levels of the following genes were determined using real-time PCR analysis: interleukin (*Il*)*-1β, Il-6, Tnfα, Il-4, Il-10, Ptp1b, Socs3,* and glyceraldehyde phosphate dehydrogenase (*Gapdh*) (Supplementary Table S1). The mRNA expression levels were normalized to that of *Gapdh*.

### 2.6 ICV injection

Stereotaxic surgery was performed on seven-week-old C57BL/6 mice anesthetized by continuous inhalation of 1.5%–2% isoflurane. Stainless steel guide cannulas (26-gauge) (P1 Technologies, Cat# C313G/SPC) were implanted into the 3V of the mice (Stereotaxic coordinates: 1.5 mm caudal to bregma and 5.5 mm ventral to the sagittal sinus). The guide cannula was attached onto the skull with dental cement (Vertex Resin Self Curing). The dummy cannula (P1 Technologies, Cat# C313DC/SPC) was inserted into the guide cannula at all times except during the time of drug injection through the internal cannula (P1 Technologies, Cat# C313I/SPC). After 7 days of recovery, 50 ng of angiotensin-2 (Sigma, Cat# A9525) was administered through each cannula to test whether the cannulas were positioned correctly. Angiotensin-2 injection by the ICV route is commonly performed to test for proper cannula placement in mice (Faesel et al., 2021). Angiotensin-2 injection induces drinking behavior within 15 min, but mice with misplaced cannulas do not display this drinking behavior. In this study, as per the previous studies (Lee et al., 2018; Kang et al., 2021), we excluded the mice that did not show a drinking response to angiotensin-2. All drugs used in ICV injections were injected at a rate of 5 μg/min using a Harvard apparatus (Cat# 70-2000) at a dose volume of 2 μl.

### 2.7 Leptin sensitivity test

The dosage and timeline of leptin used in this study were based on the previous work (Lee et al., 2018). Leptin (1 μg) was dissolved in 2 μg normal saline prior to ICV administration. Leptin was injected into the 3V on the 5^th^ day of vehicle, OLZ, and OLZ + MET administration during the light phase. Mice were cardiac-perfused at 45 min after ICV leptin injection for c-Fos immunofluorescence staining. For the leptin feeding study, leptin was injected into overnight-fasted mice. Food intake and body weight changes were monitored for 24 h post leptin injection. Plasma leptin concentrations were measured after 5 days of OLZ or OLZ + MET administration by a leptin enzyme-linked immunosorbent assay (Invitrogen, #KMC2281).

### 2.8 Statistics

All data are presented as mean ± standard error of mean (SEM). Statistical analyses were performed using Prism version 9.3.0 (GraphPad). Statistically significant differences among the groups were detected using one-way, two-way, or repeated measures analysis of variance (ANOVA) followed by a post hoc least significant difference test or a two-sided Student’s *t*-test if appropriate. Statistical significance was defined by *p* < 0.05.

## 3 Results

### 3.1 MET attenuates OLZ-induced weight gain and hyperglycemia in female mice

OLZ administration has been associated with weight gain in humans, but this phenomenon experimentally varies depending on the kind of animal and strain, sex, food, and dietary conditions (Cope et al., 2005; Davey et al., 2012; Lord et al., 2017; Zapata and Osborn, 2020). Administering OLZ to mice in conjunction with a 45% HFD is a widely used method because this mode of drug administration closely resembles that of humans (Morgan et al., 2014; Perez-Gomez et al., 2018; Zapata et al., 2020). Consistent with previous reports, we observed that OLZ administration in conjunction with a 45% HFD stimulated hyperphagia-induced weight gain in C57BL/6J female mice (Supplementary Figures S1A,B). However, as HFD itself can induce microgliosis and hyperglycemia, we investigated whether OLZ-induced side effects were observed even in animals fed a normal CD.

To test whether OLZ can independently affect weight gain, we administered OLZ to CD-fed mice. In male mice, prolonged OLZ administration did not lead to changes in food intake and body weight (Supplementary Figures S1C,D). In female mice, however, food intake was increased in 2 weeks after OLZ administration. Moreover, approximately 46% of the animals (‘obese’ group) showed delayed OLZ-induced weight gain compared to control or ‘non-obese’ groups among the female mice (Supplementary Figure S1E). Although the difference in food intake between the “obese” and “non-obese” group was not significant, more food was consumed in the “obese” group than in the control or “non-obese” groups. This might explain the weight difference between “obese” and “non-obese” groups (Supplementary Figures.S1E,F).

MET is widely prescribed for reducing the side effects of OLZ (Baptista et al., 2007; de Silva et al., 2015; de Silva et al., 2016). In animal models, MET has been reported to alleviate OLZ-induced weight gain (Hu et al., 2014; Li et al., 2018; Guo et al., 2021). We observed that MET coadministration significantly reduced weight gain in OLZ-administered female ‘obese’ mice and food intake in both the ‘non-obese’ and ‘obese’ groups (Supplementary Figures.S1E,F). It is noteworthy that MET may have a protective effect on AIWG, which may be more pronounced than the weight-loss effect. Among the male mice, the body weight and food intake in the MET group were not significantly different from those in the OLZ-alone group (Supplementary Figures S1C,D).

Previous reports have shown that systemic administration of OLZ induces hyperglycemia (Ikegami et al., 2013; Castellani et al., 2018; Boyda et al., 2022). To determine whether metabolic abnormalities precede weight gain, we conducted a glucose tolerance test after a 5-days administration of OLZ or OLZ + MET in both female and male mice. Of note, a 5-days OLZ administration significantly increased 15 and 30 min glucose level only in female mice (Figures 1B,C). In a direct comparison between OLZ-administered female and male mice, the increase rate in blood glucose compared with the baseline was significantly higher in female mice than in male mice (Supplementary Figure S2). MET significantly lowered OLZ-induced hyperglycemia in female mice and alleviated glucose levels at some time points in male mice (Figures 1B,C). These results show that MET alleviates both hyperphagia-induced obesity and hyperglycemia caused by OLZ in female mice.

### 3.2 MET attenuates OLZ-induced microgliosis and inflammation in the hypothalamus

**FIGURE 2:**
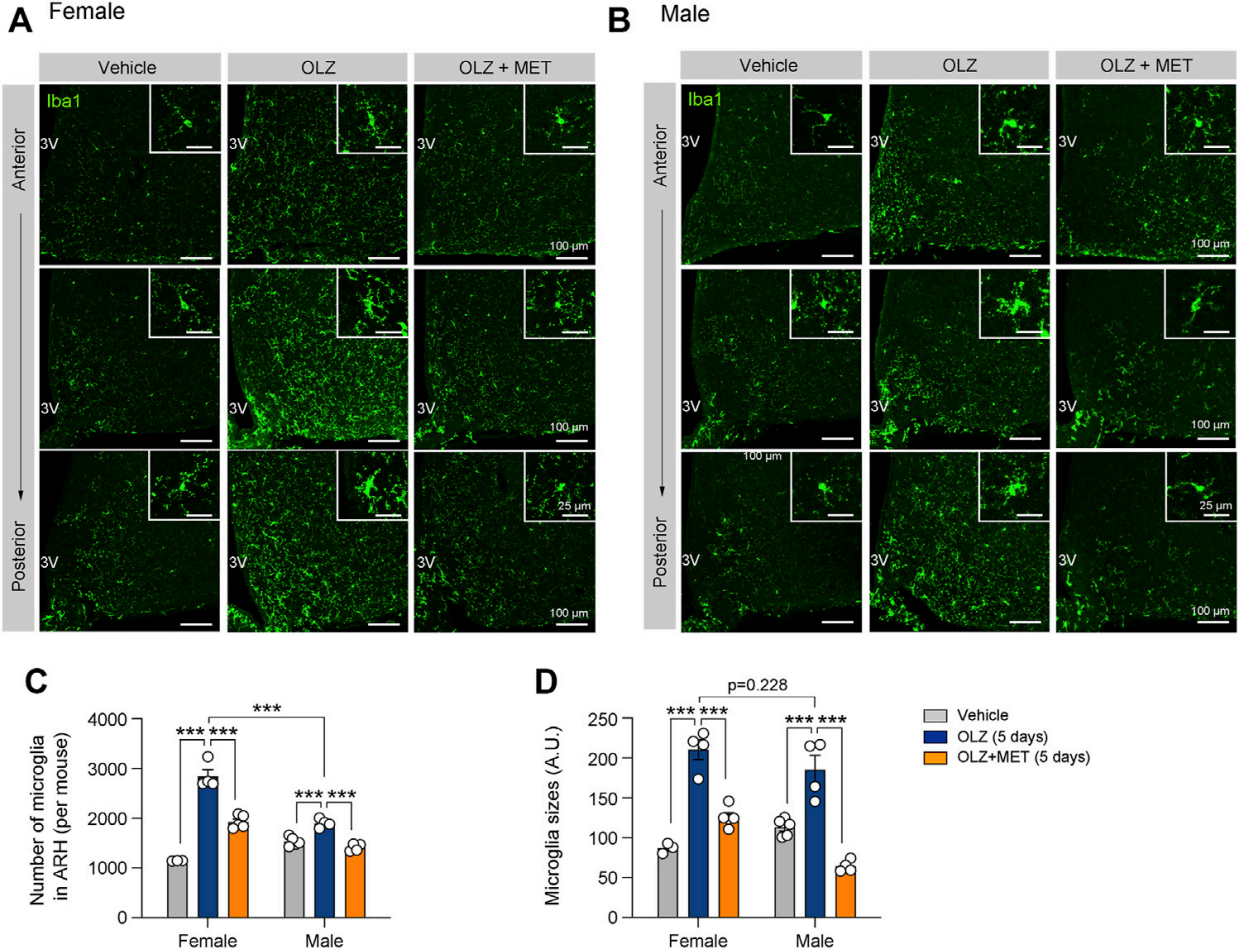
Metformin (MET) attenuates olanzapine (OLZ)-induced microglial activation in the hypothalamus. **(A,B)** Representative images of ionized calcium-binding adapter protein 1 (Iba1)-expressing microglia in the ARH of female and male mice that were administered OLZ or OLZ + MET for 5 days (*n* = 3–5). **(C,D)** The numbers and sizes of Iba1-expressing microglia in the ARH of female and male mice that were administered OLZ or OLZ + MET for 5 days (*n* = 3–5). Data are presented as mean ± SEM values. Statistical analyses were performed using one-sided one-way analysis of variance (ANOVA) followed by a post hoc least significant difference (LSD) test. ***p < 0.001 between the indicated groups. Scale bars: 100 μm.

We next investigated the mechanism by which OLZ induces hyperphagia-related obesity and glucose intolerance only in female mice. Hypothalamic inflammation and microgliosis are closely associated with metabolic dysfunctions, such as glucose intolerance and insulin resistance (Valdearcos et al., 2015; Jais and Brüning, 2017). We analyzed hypothalamic microgliosis in brain slices from mice administered OLZ or OLZ + MET for 5 days. OLZ induced a significant increase in microglial proliferation and activation in both female and male hypothalamus (Figures 2A,B). However, the microglial proliferation was far more severe in female mice than in male mice (Figures 2A–D). MET coadministration reduced hypothalamic microgliosis in both female and male mice (Figures 2A,D).

**FIGURE 3:**
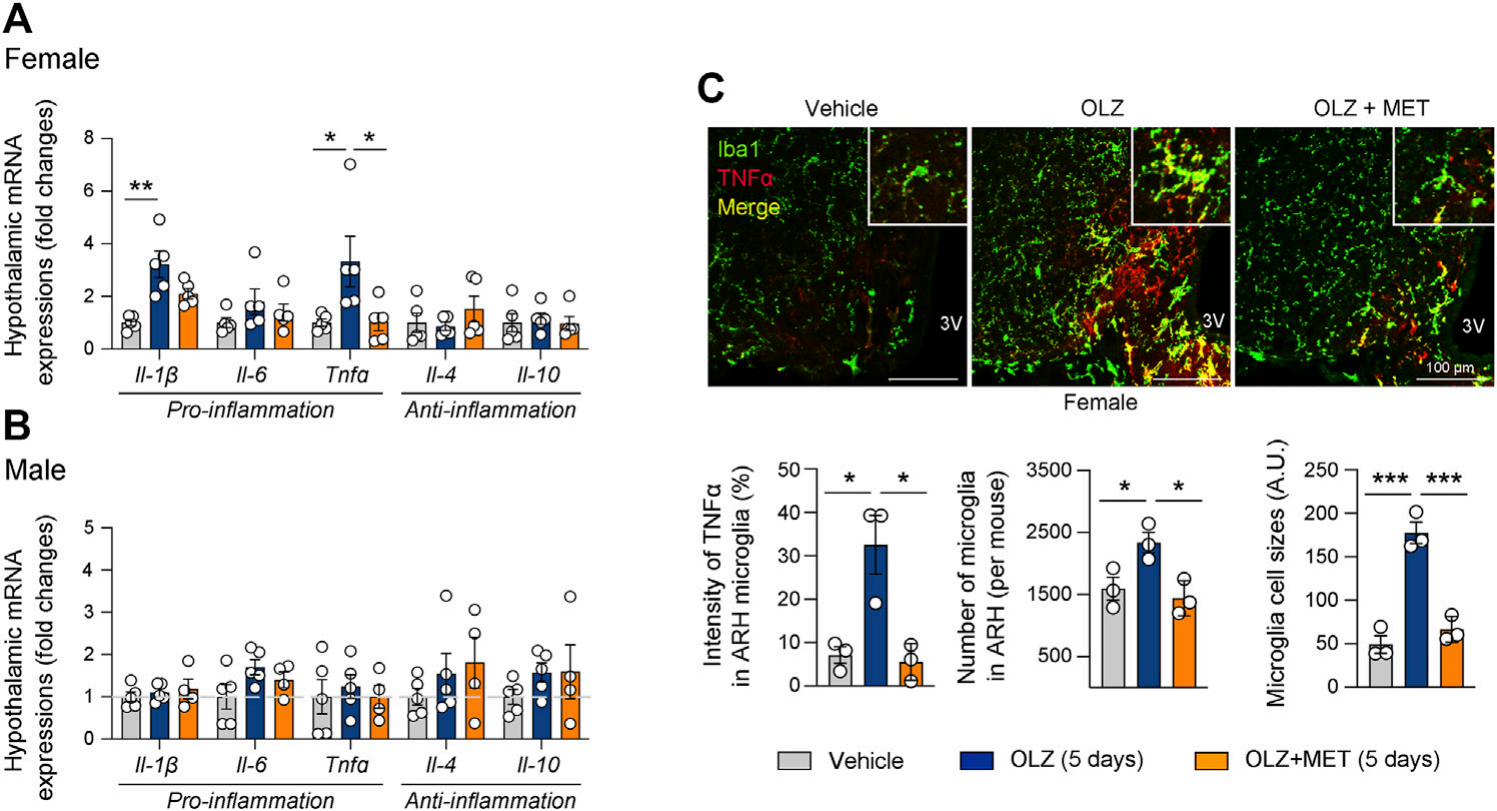
Metformin (MET) attenuates olanzapine (OLZ)-induced microglial activation and inflammation in the hypothalamus. **(A,B)** Effect of OLZ or OLZ + MET administration on the hypothalamic mRNA expression of *Il-1β*, *Il-6*, *Tnfα*, *Il-4*, and *Il-10* in female and male mice (*n* = 4–5). **(C)** Representative images of Iba1 and TNFα expression in ARH (*n* = 3). Data are presented as mean ± SEM values. Statistical analyses were performed using one-sided one-way analysis of variance (ANOVA) followed by a post hoc least significant difference (LSD) test. *p < 0.05, **p < 0.01, and ***p < 0.001 between the indicated groups. Scale bars: 100 μm.

We also measured the expression of pro-inflammatory cytokines, such as IL-1β, IL-6, and TNFα, and anti-inflammatory cytokines, such as IL-4 and IL-10 in the mediobasal hypothalamus. OLZ significantly increased the expression of pro-inflammatory cytokines only in the female hypothalamus (Figures 3A,B, and Supplementary Figure S3); this effect was reversed by MET coadministration (Figure 3A). Consistent with the results of the gene expression analysis, our immunostaining results revealed that in female mice, OLZ upregulated TNFα expression in hypothalamic microglia, which was ameliorated by MET coadministration (Figure 3C). These results suggest that MET alleviated OLZ-induced microglial activation and inflammation in the female hypothalamus.

### 3.3 Central MINO administration alleviates OLZ-induced glucose intolerance

**FIGURE 4:**
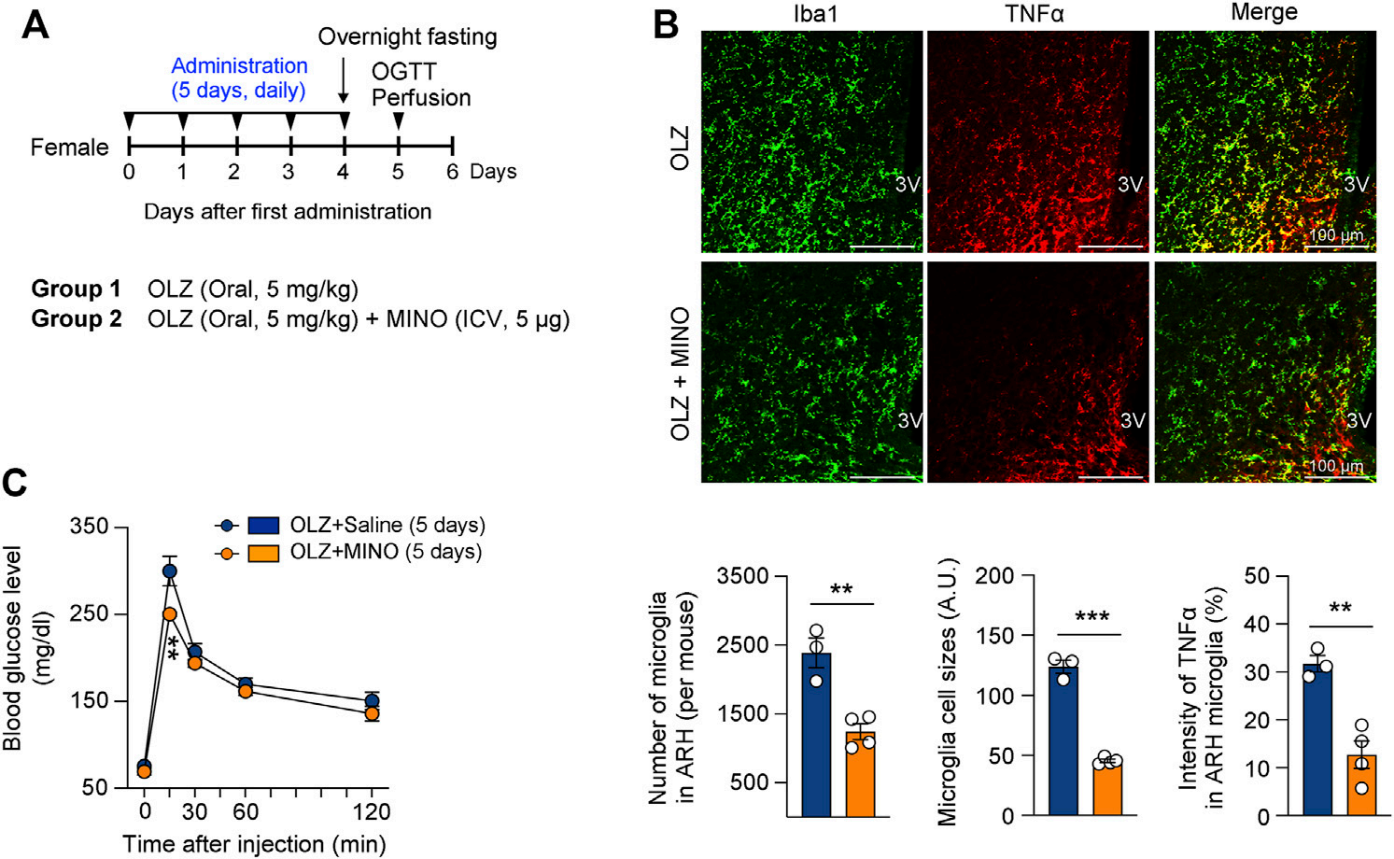
Minocycline (MINO) ameliorates olanzapine (OLZ)-induced glucose intolerance by decreasing microglial activation in female mice. **(A)** Experimental scheme pertaining to mice that were administered OLZ or OLZ + MINO on postnatal week 7. **(B)** The numbers and sizes of Iba1-expressing microglia and the expression of microglial TNFα in the ARH of female mice that were administered OLZ or OLZ + MINO for 5 days (*n* = 3–4). **(C)** Glucose tolerance tests were performed after the administration of OLZ or OLZ + MINO for 5 days (*n* = 4). Data are presented as mean ± SEM values. Statistical analyses were performed using two-sided Student’s *t-*test **(B)** and one-sided two-way analysis of variance (ANOVA) **(C)** followed by a post hoc least significant difference (LSD) test. **p < 0.01 and ***p < 0.001 between the indicated groups. Scale bars: 100 μm.

As both OLZ and MET were administered orally, it was necessary to ensure that the above-mentioned OLZ-induced phenomena were attributable primarily to hypothalamic alteration and not to the peripheral effects of the drugs. MINO is a semisynthetic tetracycline derivative that prevents microglial activation by inhibiting M1 polarization (Tikka et al., 2001; Krady et al., 2005; Kobayashi et al., 2013). Moreover, a recent study has shown that the coadministration of MINO with OLZ prevents metabolic side effects in mice (Perez-Gomez et al., 2018; Zapata et al., 2020). To demonstrate that the OLZ-induced metabolic dysfunction was due to hypothalamic microglial activation, we administered MINO daily into the 3V 1 h prior to OLZ administration (Figure 4A). The central administration of MINO not only prevented microglial activation and expansion but also decreased TNFα expression in the microglia of the hypothalamus in OLZ-administered female mice (Figure 4B). Moreover, central MINO administration alleviated OLZ-induced glucose intolerance (Figure 4C), which indicates that hypothalamic microglial activation underlies OLZ-induced systemic glucose intolerance.

### 3.4 MET and MINO prevent hypothalamic leptin resistance induced by OLZ

**FIGURE 5:**
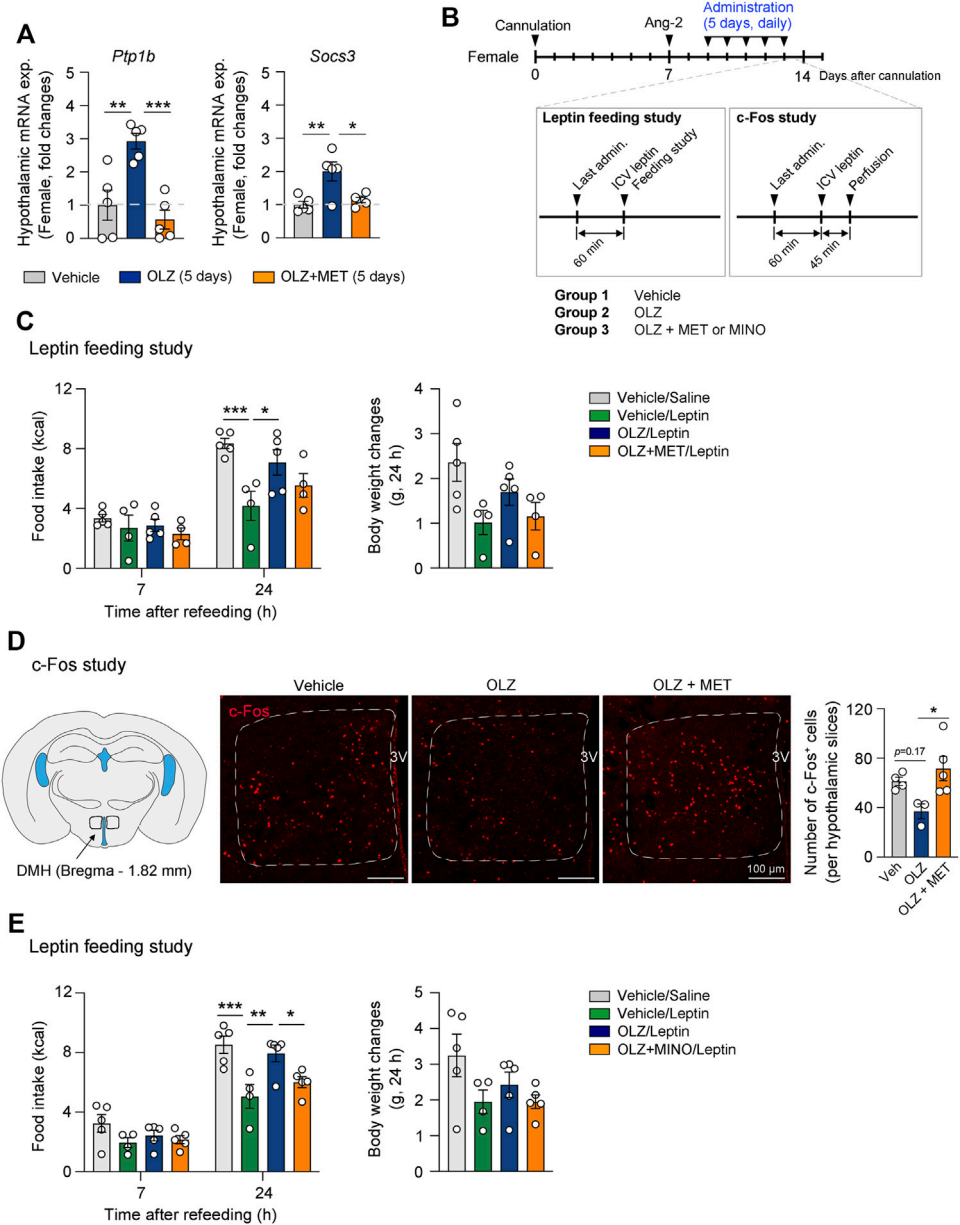
Olanzapine (OLZ)-induced leptin resistance was reversed by metformin (MET) and minocycline (MINO). **(A)** Effect of OLZ or OLZ + MET treatment on hypothalamic *Ptp1b* and *Socs3* mRNA expression in female mice (*n* = 4–5). **(B)** Experimental scheme pertaining to mice that were administered OLZ, OLZ + MET, or OLZ + MINO. **(C,E)** Food intake and body weight changes after leptin injection in female mice administered OLZ, OLZ + MET, or OLZ + MINO for 5 days (*n* = 4–5). **(D)** The numbers of c-Fos^+^ cells in the dorsomedial hypothalamus (DMH) of female mice treated with OLZ or OLZ + MET for 5 days (*n* = 3–5). Data are presented as mean ± SEM values. Statistical analyses were performed using one-sided one-way analysis of variance (ANOVA) **(A,D)** and one-sided two-way ANOVA **(C,E)** followed by a post hoc least significant difference (LSD) test. *p < 0.05, **p < 0.01, and ***p < 0.001 between the indicated groups. Scale bars: 100 μm.

Leptin is an important adipocyte-derived hormone that regulates food intake and energy expenditure (Coll et al., 2007). It is also well known that the central action of leptin is an essential component of the glucose control mechanism (Morton and Schwartz, 2011). As we found that OLZ can induce hypothalamic inflammation, dysglycemia, and weight gain, we examined whether OLZ-induced hypothalamic inflammation leads to hypothalamic leptin resistance. Studies have shown that hypothalamic inflammatory cytokines, such as IL-1β, IL-6, and TNFα, diminish leptin response by upregulating PTP1B and SOCS3, which are negative regulators of leptin signaling (Bjorbak et al., 2000; Zabolotny et al., 2002; Zabolotny et al., 2008; Ito et al., 2012; Cao et al., 2018). Thus, we first examined leptin sensitivity and further evaluated the expression of PTP1B and SOCS3 after OLZ or OLZ + MET administration. The expression levels of these molecules were significantly increased by OLZ administration and were normalized by MET coadministration in the female mouse hypothalamus (Figure 5A). These changes were not observed in the male mouse hypothalamus (Supplementary Figures S5, S6).

We next performed a leptin feeding study to examine whether the response to leptin is altered by OLZ or OLZ + MET administration (Figure 5B). Our data showed that leptin-induced anorexigenic effect was decreased by OLZ administration and that this decrease was reversed by MET coadministration (Figure 5C). Neurons in the dorsomedial hypothalamus (DMH) play a pivotal role in regulating food intake and energy expenditure (Zhang et al., 2018). Leptin administration increases the number of c-Fos + neurons at least two-fold in the DMH (Rezai-Zadeh et al., 2014). Our data showed that OLZ administration impaired leptin-induced activation of DMH neurons, and that this effect was significantly reversed by MET coadministration in the female mouse hypothalamus (Figure 5D). We next assessed whether microglial activation was responsible for OLZ-induced leptin resistance in the hypothalamus. Our feeding study data showed that central MINO administration significantly recovered leptin response in OLZ-administered female mice (Figure 5E). Inflammatory cytokines contribute to the expression of SOCS3 and PTP1B, which leads to leptin resistance by blocking leptin receptor signaling (Zabolotny et al., 2008; Ito et al., 2012; Cao et al., 2018). There was no significant change in the plasma leptin concentration after the administration of OLZ or OLZ + MET for 5 days (Supplementary Figures S7). These data suggest that OLZ-induced leptin resistance primarily arises from OLZ-induced hypothalamic microglial activation.

## 4 Discussion

In this study, we investigated the mechanism by which OLZ induces obesity and metabolic disorders. Despite the importance of hypothalamic microgliosis and inflammation in the development of metabolic disorders, the relationship between OLZ and hypothalamic microgliosis has remained unclear. In the present study, we discovered sex-dependent differences in response to OLZ administration. We found that OLZ-induced hypothalamic microgliosis, which plays a pivotal role in the development of metabolic disorders and obesity, was more pronounced in female mice than in male mice. Moreover, our findings indicated that MET, a drug that is commonly used for ameliorating the metabolic side effects of OLZ, also exerts its effect by regulating hypothalamic microgliosis.

We have focused on OLZ-induced microgliosis in accordance with previous studies reporting that hypothalamic inflammation and microgliosis are major causes of obesity, hyperphagia, and glucose intolerance (Valdearcos et al., 2017; Lee et al., 2018). Recently, it was reported that the pathogenesis of OLZ-induced obesity is associated with hypothalamic inflammation induced by toll-like receptor-4 signaling in astrocytes (another type of glial cell in the central nervous system) (He et al., 2020). In this study, astrogliosis was observed in the OLZ-administered pair-fed group, suggesting that OLZ directly stimulates hypothalamic glial cells (He et al., 2020). These findings, similar to our study, suggest the essential role of hypothalamic inflammation in the pathogenesis of OLZ-induced metabolic side-effects.

Mouse studies on AP-induced side effects have shown that female mice (C57BL/6J) are more susceptible to OLZ-induced weight gain and metabolic disturbances than male mice (Fernø et al., 2011; Lord et al., 2017; Zapata et al., 2021). This is also in accordance with clinical data showing that female patients are more susceptible to weight gain and dysglycemia than male patients (Bobes et al., 2003; McEvoy et al., 2005; Iversen et al., 2018). In our experiment, we compared hypothalamic microglial changes between male and female mice. Female mice showed a significant increase in hypothalamic microglia, whereas male mice showed a much smaller increase, which could explain why male mice did not present an increase in inflammatory cytokines and OLZ-induced weight gain. This suggests that hypothalamic inflammation plays a key role in OLZ-induced metabolic side effects. We further examined the role and implication of hypothalamic inflammation in AIWG. We found that MET, a commonly used medication for AIWG, can ameliorate OLZ-induced hypothalamic inflammation. We also found that OLZ induces hypothalamic leptin resistance *via* the elevation of *Socs3* and *Ptp1b* expression; these genes are directly related to hypothalamic inflammation and microglial activation (Zabolotny et al., 2008; Ito et al., 2012; Cao et al., 2018).

However, several aspects remain to be clarified. In our experiment, some OLZ-administrated CD-fed mice did not show OLZ-induced weight gain (OLZ-administered “non-obese” group, Supplementary Figures S1E,F). There was no significant difference in body weight between these mice and the control mice at the end of the study. In contrast, several mice showed significant weight gain under the same conditions (OLZ-administered “obese” group). This can be partially explained by differences in food intake (Supplementary Figure S1F), which is consistent with the current knowledge that OLZ-induced weight gain is mediated by hyperphagia (Fernø et al., 2011; Lord et al., 2017; Perez-Gomez et al., 2018; Zapata et al., 2021). Of note, significant hypothalamic microgliosis and hyperglycemia were observed in all female mice. One possible explanation is that hypothalamic inflammation in the arcuate nucleus of the hypothalamus (ARH), although necessary, is not sufficient for the onset of AIWG. Other conditions that are induced by HFD might be necessary for the increase in food intake or onset of obesity (Supplementary Figures S1A,B).

A recent study provided insight into the above-mentioned aspect (Zapata et al., 2021). In that study, OLZ-administered HFD-fed mice showed changes in weight gain, which was explained by variations in the immunomodulatory transcription network (Zapata et al., 2021). Given these data, it would be reasonable to assume that the interplay between activated microglia and changes in transcription factors caused by various conditions is necessary for OLZ-induced hyperphagia. Our data also helped elucidate the mechanism of action of MET, a commonly used type 2 diabetes mellitus and AIWG medication.

**FIGURE 6:**
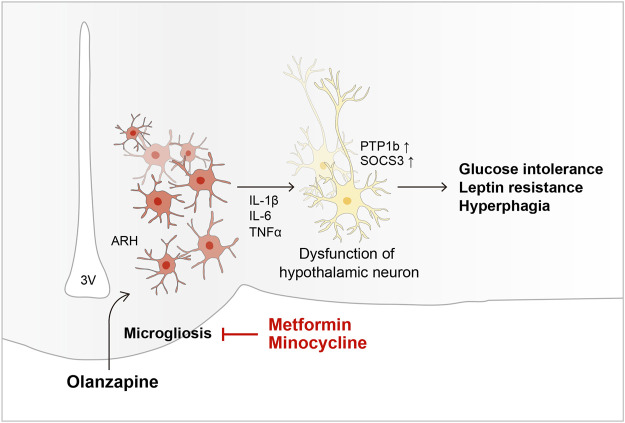
Schematic diagram of the mechanism underlying OLZ-induced hypothalamic microgliosis and metabolic dysfunction.

To summarize, our findings showed that OLZ independently induces hypothalamic microglial activation, and that this effect is necessary for OLZ-induced weight gain and dysglycemia (Figure 6). We also showed that MET, a commonly used AIWG medication, exerts its effect by regulating hypothalamic microglial actions. Further research on the sex-related differences in the side effects of OLZ and the role of microglia in the course of obesity development is required. Moreover, the American Diabetes Association recently announced the rising importance of glucagon-like peptide-1 and sodium-glucose cotransporter-2 inhibitors to treat type 2 diabetes and its associated complications (American Diabetes Association, 2022); additional studies investigating the effects of these drugs are required. Taken together, these data will contribute to the development of APs with fewer side effects or medications that effectively prevent AIWG and hyperglycemia.

## Data Availability

The original contributions presented in the study are included in the article/Supplementary Material, further inquiries can be directed to the corresponding authors.

## Abbreviations

OLZ, olanzapine; MET, metformin; AIWG, antipsychotic drug-induced weight gain; PTP1B, protein tyrosine phosphatase 1B; SOCS3, suppressor of cytokine signaling 3; HFD, high-fat diet; MINO, minocycline; Iba1, ionized calcium-binding adaptor protein-1; TNF, tumor necrosis factor; IL, Interleukin; CD, chow diet; DMH, dorsomedial hypothalamus; APs, antipsychotics; SEM, standard error of mean; 3V, 3rd ventricle; ANOVA, analysis of variance; ADA, American Diabetes Association; GLP-1, glucagon-like peptide-1; SGLT-2, sodium-glucose cotransporter-2.
