# Supplementary material for: Metformin ameliorates olanzapine-induced obesity and glucose intolerance by regulating hypothalamic inflammation and microglial activation in female mice
Source: Front Pharmacol. 2022 Oct 12;13:906717. doi: 10.3389/fphar.2022.906717 (PMC9596779; doi:10.3389/fphar.2022.906717)
Supplement: Supplementary file 1 [file Table1.DOCX]

Supplementary Material

# Supplementary Figures and Tables

## Supplementary Figures

**
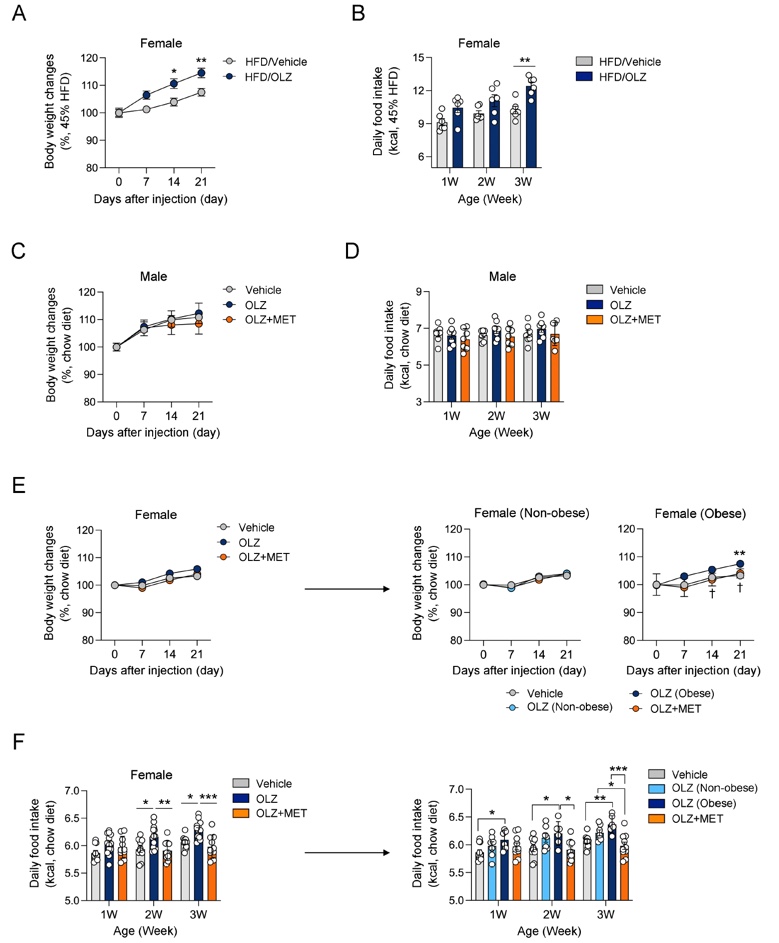
**

**Supplementary Figure 1.** **Metformin (MET) attenuates hyperphagia-based weight gain induced by olanzapine (OLZ) in female mice**

**A, B** Body weights and daily food intake of high-fat (45% fat) diet-fed female mice that were administered OLZ (n = 6). **C, D** Body weights and daily food intake of male mice that were administered OLZ or OLZ+MET (n = 7). **E, F** Body weights and daily food intake of female mice that were administered OLZ or OLZ+MET (n = 10‒13). The graphs on the right of the arrow are graphs dividing the OLZ group into ‘non-obese’ and ‘obese’ groups. Data are presented as mean ± SEM values. Statistical analyses were performed using one-sided two-way analysis of variance (ANOVA) followed by a post hoc least significant difference (LSD) test. **p* < 0.05, ***p* < 0.01 and ****p* < 0.001 between the indicated groups.


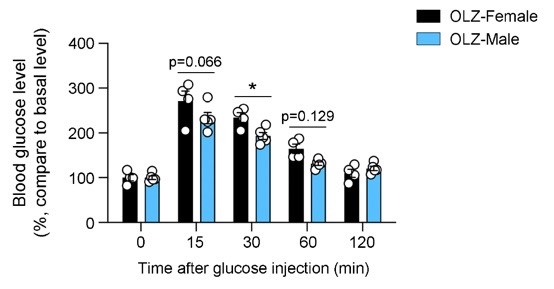


**Supplementary Figure 2. Comparison of blood glucose changes between OLZ-administered female and male mice.** Data are presented as mean ± SEM values. Statistical analyses were performed using one-sided two-way analysis of variance (ANOVA) followed by a post hoc least significant difference (LSD) test. **p* < 0.05 between the indicated groups.


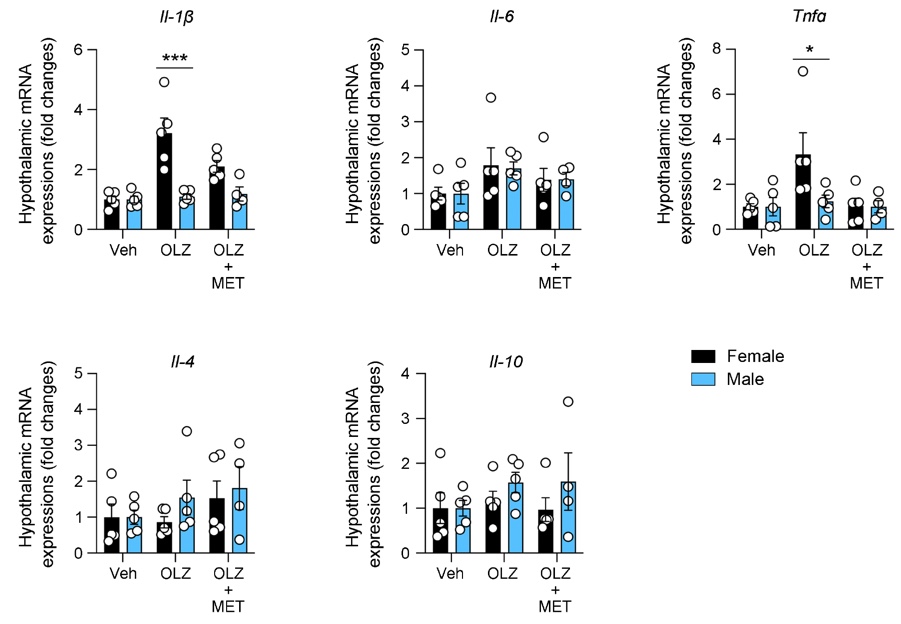


**Supplementary Figure 3. Comparison of mRNA expression of hypothalamic inflammatory cytokines between female and male mice.** Data are presented as mean ± SEM values. Statistical analyses were performed using one-sided two-way analysis of variance (ANOVA) followed by a post hoc least significant difference (LSD) test. **p* < 0.05 and ****p* < 0.001 between the indicated groups.


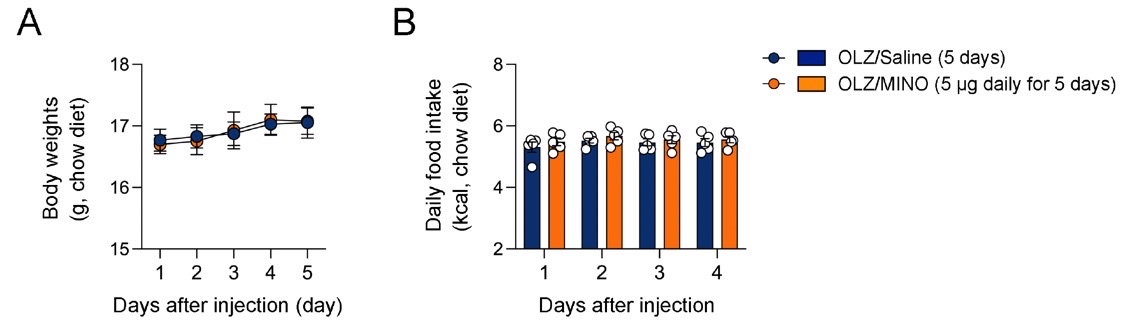


**Supplementary Figure 4. Daily minocycline (MINO) (5 μg) administration for five days did not alter body weight and daily food intake**

**A, B** Body weights and daily food intake of OLZ-administered female mice that were administered MINO by intracerebroventricular injection (n = 5). Data are presented as mean ± SEM values. Statistical analyses were performed using one-sided two-way analysis of variance (ANOVA) followed by post hoc least significant difference (LSD) test.

**
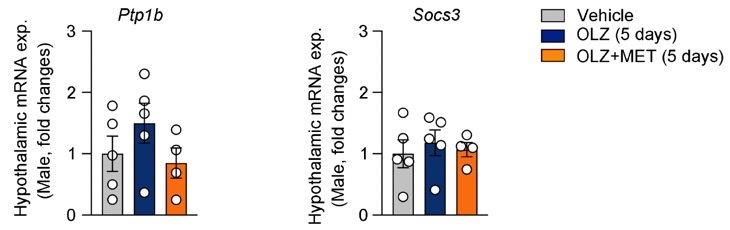
**

**Supplementary Figure 5. Effect of OLZ or OLZ+ MET treatment on hypothalamic *Ptp1b* and *Socs3* mRNA expression in male mice**.

Data are presented as mean ± SEM values. Statistical analyses were performed using one-sided one-way analysis of variance (ANOVA) followed by a post hoc least significant difference (LSD) test.


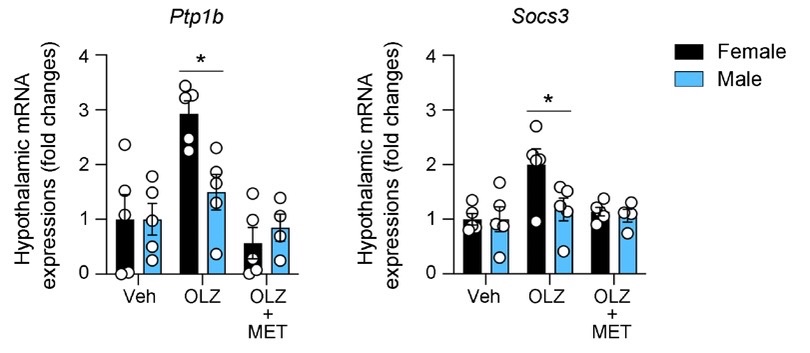


**Supplementary Figure 6. Comparison of mRNA expression of *Ptp1b* and *Socs3* between female and male mouse hypothalamus.** Data are presented as mean ± SEM values. Statistical analyses were performed using one-sided two-way analysis of variance (ANOVA) followed by a post hoc least significant difference (LSD) test. **p* < 0.05 between the indicated groups.


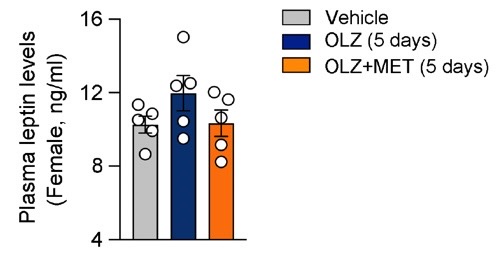


**Supplementary Figure 7. Plasma leptin concentrations of female mice that were administered OLZ or OLZ+MET for five days.**

Data are presented as mean ± SEM values. Statistical analyses were performed using one-sided one-way analysis of variance (ANOVA) followed by a post hoc least significant difference (LSD) test.

## Supplementary Table

**
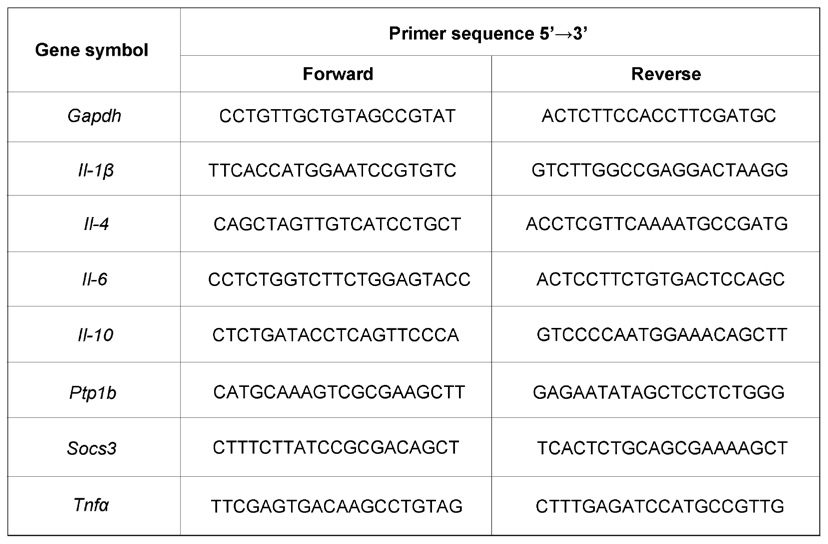
**

**Supplementary Table 1. Primers used for the analysis of gene expression by real-time PCR in this study**
